# Update on the management of acute respiratory failure using non-invasive ventilation and pulse oximetry

**DOI:** 10.1186/s13054-023-04370-4

**Published:** 2023-03-21

**Authors:** Tatsuhiko Abe, Toshishige Takagi, Tomoko Fujii

**Affiliations:** Intensive Care Unit, Jikei University Hospital, Tokyo, Japan

## Abstract

This article is one of ten reviews selected from the Annual Update in Intensive Care and Emergency Medicine 2023. Other selected articles can be found online at https://www.biomedcentral.com/collections/annualupdate2023. Further information about the Annual Update in Intensive Care and Emergency Medicine is available from https://link.springer.com/bookseries/8901.

## Introduction

The coronavirus disease 2019 (COVID-19) pandemic has been the biggest challenge to intensive care providers globally since the invention of ventilators in the 1930s to support patients with polio [1]. The huge number of patients with COVID-19 urged us to investigate better and safer strategies for managing respiratory failure. Given the limited resources for the large number of patients presenting with acute respiratory failure, we used non-invasive devices, i.e., pulse oximeters and non-invasive ventilation (NIV), to monitor and manage patients outside the ICU. This raised attention regarding the uncertainties of using such devices in the management of critically ill patients.

NIV emerged as a respiratory support system to reduce the need for endotracheal intubation and the risk of death. More patients than ever received respiratory support with NIV during the COVID-19 pandemic, in part also because of the limited availability of invasive mechanical ventilation. Clinicians and researchers have attempted to determine the optimal management of respiratory failure using NIV. In this review, we summarize recent clinical research findings on the management of acute respiratory failure using NIV and pulse oximetry. For the purposes of this article, NIV includes non-invasive positive pressure ventilation (NPPV) and high-flow nasal cannula oxygen (HFNC).

## Effectiveness and utility of NIV

In their systematic review published in 2020, Ferreyro et al. reported that NPPV was associated with a lower risk of mortality, particularly with helmet type (risk ratio (RR) 0.40 [95% CI 0.24–0.63]), and also with face mask type (RR 0.83 [95% CI 0.68–0.99]) in acute hypoxemic respiratory failure [2]. NPPV was also associated with lower risks of intubation (helmet type, RR 0.26 [95% CI 0.14–0.46], face mask type, RR 0.76 [95% CI 0.62–0.90]) when compared with conventional oxygen therapy. In contrast, HFNC was not significantly associated with a lower risk of mortality (RR 0.87 [95% CI 0.62–1.15]); however, it was associated with a reduced risk of intubation (RR 0.76 [95% CI 0.55–0.99]). Thus, the best probability of reducing all-cause mortality and intubation was with helmet NPPV.

Another systematic review, published in 2021, evaluated ventilation modes in addition to respiratory support devices and compared continuous positive airway pressure (CPAP), pressure support ventilation (PSV), HFNC, and conventional oxygen therapy using a network meta-analysis [3]. Only CPAP was associated with improved mortality compared to conventional oxygen therapy (RR 0.55 [95% CI 0.31–0.95]), and CPAP and PSV were associated with a lower risk of intubation (CPAP, RR 0.48 [95% CI 0.30–0.79]; PSV RR 0.67 [95% CI 0.51–0.89]). HFNC was not associated with a significantly lower risk of mortality or intubation. Thus, the best probability of reducing all-cause mortality and intubation was with CPAP.

### Key clinical trials from the systematic reviews

Frat et al. conducted a randomized clinical trial (RCT) to assess the efficacy of HFNC for acute hypoxemic respiratory failure compared to conventional oxygen therapy and NPPV [4]. The trial revealed that intubation at 28 days was not significantly different between HFNC (38% [40/106]), conventional oxygen therapy (47% [44/94]), and NPPV (50% [55/110]) (p for the three-group comparison = 0.18). However, the risk of death at 90 days was significantly higher with conventional oxygen therapy (hazard ratio (HR) 2.01 [95% CI 1.01–3.99]) and NPPV (HR 2.50 [95% CI 1.31–4.78]) compared to HFNC. HFNC might reduce mortality at 90 days because the large tidal volume in the NPPV group (median 9.2 ± 3.0 ml/kg) may lead to *de novo* lung injury (Table 1).

**Table 1:** Summary of key randomized clinical trials of non-invasive ventilation (NIV) and high-flow nasal cannula oxygen (HFNC)

Study	No. of patients	Cause of AHRF	P/F	Interventions (cmH_2_O)	V_T_ (ml/ kg)	Comparator	Primary outcome	Results	Time to intubation
Frat [4]	310	CAP (63.5%)	155	NPPV-PS, face mask, PS 5, PEEP 8	9.2	HFNC, COT	Intubation at 28 day	38% with HFNC, 47% with COT, and 50% with NPPV (p = 0.18 for all comparisons)	23 h
Lemiale [5]	374	Pneumonia (68.7%)	142	NPPV-PS, face mask, PS 2–10, PEEP N/A	9.1	COT	Mortality at 28 day	24.1% vs. 27.3%, p = 0.47	Half of the intubation events occurred in 1 day
Azoulay [6]	776	Pneumonia (77.6%)	132	HFNC	−	COT	Mortality at 28 day	35.6% vs. 36.1%, p = 0.94	Half of the intubation events occurred in 1 day
He [7]	200	CAP (94%)	231	NPPV-PS, face mask, PS 6, PEEP 8	8.1	COT	Intubation	10.8% vs. 9.2%, p = 0.72	3.65 days
Coudroy [8]	299	Pneumonia (74.5%)	147	NPPV-PS alternating with HFNC, face mask, PS 7, PEEP 7	9.6	HFNC alone	Mortality at 28 day	35% vs. 36%, p = 0.83	24 h
Ospina- Tascon [9]	220	COVID-19	104	HFNC	−	COT	Intubation within 28 days	34.3% vs. 51.0%, HR 0.62 (0.39–0.96), p = 0.03	25.75 h
Perkins [10]	1273	COVID-19	114	NPPV-CPAP, mostly face mask, PS 8.3, PEEP N/A	N/A	COT	Intubation or mortality within 30 days	36.3% vs. 44.4%, p = 0.02	1.5 days
				HFNC	−	COT	Intubation or mortality within 30 days	44.3% vs. 45.1%, p = 0.69	1 day

*AHRF* acute hypoxemic respiratory failure, *P/F* PaO_2_ FiO_2_ ratio, *V*_*T*_ tidal volume, *COT* conventional oxygen therapy, *NPPV* non-invasive positive pressure ventilation, *PEEP* positive end-expiratory pressure, *CPAP* continuous positive airway pressure, *PS* pressure support, *CAP* community-acquired pneumonia, *COVID* coronavirus disease, *HR* hazard ratio

NIV is preferred in immunocompromised patients as it may mitigate the risk of ventilator-associated pneumonia (VAP). Several trials have been conducted in immunocompromised patients. One trial compared NPPV vs. conventional oxygen therapy (n = 374), and another compared HFNC vs. conventional oxygen therapy (n = 776); neither trial found any significant difference in mortality or intubation rates at 28 days. Given that the meta-analysis suggested clinical benefits of NPPV and HFNC in non-restricted patient populations [2], Coudroy et al. conducted a multicenter RCT comparing NPPV interspaced with HFNC during the interruption period to prolong the duration of respiratory support with HFNC alone in 300 adult immunocompromised patients (50% had hematological malignancy) [8]. The results, published in 2022, showed that mortality rates at day 28 did not differ between the two groups (35% [51/145] in the NIV + HFNC group, 36% [56/154] in the HFNC alone group, p = 0.83).

With preserved spontaneous breathing, a mechanism called patient self-inflicted lung injury (p-SILI) can cause further alveolar damage [11, 12]. In lungs affected by the acute respiratory distress syndrome (ARDS), typified by dorsal collapse and ventral hyperinflation, strong inspiratory efforts would cause greater local strain, damaging the alveoli histologically and physiologically. In addition, some studies have suggested that a tidal volume of 9 ml/kg or more could increase the probability of NIV failure [4, 13], which may explain the lack of benefit in these trials [4, 5, 8, 13].

## Current knowledge of NIV for respiratory failure in the ICU

In respiratory failure requiring intensive care unit (ICU) admission, NPPV or HFNC probably reduce the risk of death or intubation compared to conventional oxygen therapy. In particular, NPPV has a well-established effect on acute exacerbations of chronic obstructive pulmonary disease (COPD) and cardiogenic pulmonary edema [14–16]. Additionally, the European Respiratory Society recommends using HFNC for acute hypoxemic respiratory failure in its recent clinical practice guideline [17]. NPPV can be effective if it manages to prevent increases in inspiratory effort and tidal volume while ensuring tolerance. When managing acute hypoxemic respira- tory failure with NPPV, the ventilation mode may need attention to minimize the risk of p-SILI.

However, more information is required to inform clinical practice on how to manage acute hypoxemic respiratory failure using NIV. First, it is unclear how differences in the NPPV interface may affect clinical outcomes. For example, helmet NPPV might be more comfortable, and air leaks may be minimized. However, the most recent systematic review, which included 16 RCTs and 8 observational studies, found that the mortality benefit of helmet NPPV over face mask NPPV was of low certainty despite the statistically significant effect estimates (RR 0.56 [95% CI 0.33–0.95]) [18]. Furthermore, the effect of helmet NPPV compared to HFNC was uncertain [18]. Therefore, further well-designed and adequately powered clinical trials are needed to determine the optimal intervention with NIV. Second, preservation of spontaneous breathing may worsen existing lung injury and lead to p-SILI. However, there is not sufficient evidence on its clinical significance.

## NIV for COVID-19

The COVID-19 pandemic has forced the use of NIV outside the ICU, such as in general wards [19]. HFNC was also used for acute hypoxemic respiratory failure related to COVID-19 worldwide, even before its efficacy and safety were established [20]. A recent systematic review identified nine observational studies and only one RCT that compared HFNC and NPPV by June 2021. Due to the paucity of adequately powered RCTs, it would be premature to conclude whether NIV is clinically beneficial for patients with COVID-19 or if HFNC or NPPV is superior to the other.

### Key clinical trials and Recovery-RS

The HiFLo-Covid trial in Colombia (n = 220) compared HFNC and conventional oxygen therapy for respiratory failure due to COVID-19. HFNC was shown to reduce the rate of intubation at 28 days (34.3% vs. 51.0%, HR 0.62 [95% CI 0.39–0.96]) [9]. HFNC may reduce respiratory workload and improve gas exchange, leading to the improved intubation rate. However, physiological parameters, such as transpulmonary pressures or tidal volumes, which support the mechanisms of HFNC, were not available in this trial.

The HENIVOT trial was a RCT conducted in four ICUs in Italy (n = 110) comparing helmet NPPV and HFNC for respiratory failure in COVID-19 [21]. There was no statistically significant difference in the primary outcome, 28-day mechanical respiratory support free-days (20 days vs. 18 days, median difference (MD), 2 days, [95% CI, −2 to 6]); however, one of the secondary outcomes, the 28-day intubation rate, was reduced with helmet NPPV compared to HFNC (30% vs. 51%, HR 0.41 [95% CI 0.18–0.89]). Given the small sample sizes of the two trials, the available evidence is insufficient to conclude a positive effect of NIV or futility in patients with COVID-19.

In 2022, an awaited trial result was published. The RECOVERY-RS trial is the largest RCT of NIV strategy for respiratory failure due to COVID-19 [10]. RECOVERY-RS was a randomized, 3-arm study that compared HFNC, CPAP, and conventional oxygen therapy. Although it was terminated early, in May 2021 when 1278 patients had been enrolled, due to the decrease in numbers of hospitalized patients with COVID-19, the results showed that CPAP reduced intubation or death within 30 days compared to conventional oxygen therapy (36.3% vs. 44.4%, absolute difference −8% [95% CI −15% to −1%]). In contrast, HFNC was not significantly different compared to conventional oxygen therapy (44.3% vs. 45.1%, absolute difference −1% [95% CI −8% to 6%]). Although early termination of a trial could provide a risk of bias that overestimates the effect in general, the decision was made solely by the trial committee, which was blinded to the result of an interim analysis, thus mitigating the risk. Regrettably, the early termination made the trial underpowered to detect any clinically meaningful difference.

## Current knowledge about use of NIV for COVID-19

Schmid et al. [22] updated a systematic review and meta-analysis and analyzed three RCTs comparing HFNC to NPPV, including RECOVERY-RS. The result showed that HFNC use may increase the composite outcome of intubation or mortality rate (RR 1.22 [95% CI 1.03–1.45]). However, the available evidence was insufficient to conclude the effectiveness of NPPV or HFNC because of the low level of evidence and possible bias.

Furthermore, extra considerations are needed in managing COVID-19 respiratory failure: the risk of virus transmission through aerosol dispersed from the equipment, depletion of medical resources due to pandemics, changes in standard of care, and viral mutations [23].

## Timing of intubation when patients are receiving NIV

Delayed intubation has a poor prognosis for patients who need invasive ventilatory management. Kang et al. conducted an observational study to describe the characteristics of patients requiring tracheal intubation after HFNC failure [24]. They reported that patients who had intubation after 48 h had a higher mortality rate than those who had intubation within 48 h (66.7% [30/45] vs. 39.2% [51/130], p = 0.001).

Kangelaris et al. reported that 34% of patients with respiratory failure in multi-center ICUs who were managed without intubation at the time of meeting criteria for ARDS (PaO_2_/FiO_2_ < 300 or SpO_2_/FiO_2_ < 315) required intubation within the next 3 days [25]. Sixty-day mortality of the late intubation group was higher than that of patients intubated early (56% [20/36] vs. 36% [128/351]).

A recent systematic review, published in 2021, assessed whether early intubation of patients with COVID-19 was effective [26]. Twelve observational studies, representing 8944 patients with COVID-19, were included. In the pooled analysis there was no significant difference in mortality among those who had intubation early (within 24 h of ICU admission) and those intubated late (45.4% vs. 39.1%, p = 0.08).

The British Thoracic Society recommends evaluating patients 4–6 h after initiating NIV [27]. However, current clinical practice seems more conservative. RCTs that have assessed the effects of NIV reported that most intubation events occurred around 1–2 days after the initiation of NIV [4–6, 8]. As there are not sufficient data on the optimal timing of intubation for patients receiving NIV, close monitoring of respiratory workload and gas exchange are vital in these patients. Furthermore, optimal parameters that can inform clinicians about the best timing for intubation have yet to be determined.

## Predicting successful treatment with HFNC

A strong interest has developed into predicting treatment success for HFNC. To avoid delaying intubation, Roca et al. proposed the ratio of oxygen saturation to respiratory rate (ROX) index as an early predictor of HFNC treatment failure so that intubation can be performed in a timely manner [28, 29]. The ROX index is calculated as (SpO_2_/FiO_2_)/respiratory rate. The numerator (SpO_2_/FiO_2_) is a measure of oxygenation that is positively correlated with successful high-flow oxygen therapy. The denominator (respiratory rate) is inversely correlated to high-flow oxygen therapy success. Although the predictive performance of the ROX index has been increasingly studied in recent years, there are still only a few high-quality studies [30–32].

A systematic review of eight observational studies of COVID-19-associated pneumonia found that the summary area under the curve (SAUC) of the ROX-index to predict high-flow oxygen therapy failure was 0.81 (95% CI 0.77–0.84), with a sensitivity of 0.70 (95% CI 0.59–0.80) and specificity of 0.79 (95% CI 0.67–0.88) [33]. HFNC failure was defined as the use of either invasive or non-invasive mechan- ical ventilation. The results indicated that the ROX index had good discriminatory power to predict HFNC failure [33]. In a subgroup analysis in which only studies that examined the ROX index within 6 h of HFNC initiation were considered, there was no change in predictive accuracy [33].

Another subgroup analysis looked at the cut-off value of the ROX index. Studies with an ROX index cut-off value greater than 5 had higher predictive accuracy (SAUC, 0.87 [0.83, 0.89]) than those with a cut-off value of 5 or less (SAUC, 0.76 [0.72, 0.80]) [33].

## ROX index in COVID-19

The utility of the ROX index in COVID-19 patients was explored in another systematic review of eight observational studies and one RCT [34]. The causes of respira- tory failure were categorized as COVID-19-related pneumonia in four studies, pneumonia in two studies, hypoxic respiratory failure mainly due to pneumonia in two studies, and respiratory failure in immunocompromised patients in one study. The meta-analysis showed that the ROX index had moderate accuracy and, in a subgroup analysis, that it had higher predictive accuracy in studies of COVID-19- associated pneumonia than in studies of other diseases (COVID-19, AUC, 0.78 [0.74, 0.82]; others, 0.72 [0.68, 0.76]) [34].

The most recently updated systematic review of 13 observational studies, of which 10 included COVID-19-related pneumonia and 3 included pneumonia, found similar findings [35]. Successful withdrawal from high-flow oxygen therapy was accurately predicted by the ROX index measured within 12 h of its initiation with an area under the hierarchical summary receiver operating characteristic curve (AUHSROC) of 0.81 (95% CI, 0.77–0.84), a diagnostic odds ratio of 8.3 (95% CI 6.4–10.8), a sensitivity of 0.71 (95% CI 0.64–0.78), and a specificity of 0.78 (95% CI 0.70–0.84). The mean cut-off value was 4.8 (95% CI 4.2–5.4) and, in subgroup analysis, the predictive accuracy was high in COVID-19-related pneumonia, as previously reported [34]. Assessing the ROX index within 6 h or 6–12 h was associated with similarly good predictive ability [35].

The three systematic reviews [33–35] suggest that the ROX index measured within 12 h of initiation is a useful predictor of high-flow oxygen therapy success. However, the overall quality of the included studies was limited, and the heterogeneity observed in the pooled effects would need further exploration. Furthermore, the utility of the ROX index for NPPV is unknown as it has been developed and vali- dated only with HFNC.

## Monitoring respiratory failure using SpO_2_: the risk of inaccuracy

Dr. Takuo Aoyagi, a Japanese medical engineering specialist, invented the pulse oximeter in 1974 [36]. Since pulse oximeters use light absorption to estimate the SaO_2_, it was acknowledged early that skin color might affect the readings [37].

Jubran et al. conducted a prospective observational study of 54 ventilator-assisted patients (29 black and 25 white) at two USA institutions in 1990 [38]. In this study, the FiO_2_ of the ventilator was adjusted, and the SpO_2_ and arterial partial pressure of oxygen (PaO_2_) were recorded. The results suggested that pulse oximeters missed hypoxemia twice as often in black subjects than in white subjects. The value of SpO_2_ required to maintain PaO_2_ > 60 mmHg varied depending on the skin color. Based on an early experiment showing that the use of black nail polish reduced the difference in absorbance of red and infrared lights, and caused the pulse oximeter to falsely record a lower oxygen saturation, the disparity in the accuracy of SpO_2_ could be attributable to skin color [39]. However, this issue of measurement errors in pulse oximeters has not been pursued or sufficiently investigated until recently.

## Inaccuracy of pulse oximeters and skin color: new investigations

Since the COVID-19 pandemic outbreak in 2020, measurement errors from pulse oximeters have been revisited and studied worldwide. Sjoding et al. conducted an observational study in the USA to determine the frequency of potential hypoxemia (with SaO_2_ < 88% despite a pulse oximeter SpO_2_ of 92–96%) in 10,001 individuals (8675 white and 1326 black) [40]. Latent hypoxemia undetected by the pulse oximeter occurred about three times more frequently in black subjects than in white subjects, suggesting that pulse oximeters are unreliable for triaging patients and adjusting oxygen levels. The study was published in 2020 and received worldwide attention, especially because of its important potential implications in the midst of the COVID-19 pandemic.

In 2021, Wong et al. conducted a large retrospective observational study using an American database of 87,971 individuals, including Asians, blacks, Hispanics, and whites. They reported the prevalence of potential hypoxemia (SpO_2_ > 88% but SaO_2_ < 88%) as well as clinical outcomes [41]. With more than 80,000 participants, the precision of the study increased compared to that in the smaller study by Sjoding et al. [40]. Potential hypoxemia occurred in all racial subgroups, but there were racial differences in prevalence, being present in 6.8% of blacks, 6.0% of Hispanics, 4.8% of Asians, and 4.9% of whites (p < 0.001) [41]. These authors also noted a greater variability in SaO_2_ for any given SpO_2_ value in black subjects and reported that the risk of potential hypoxemia at 92% SpO_2_ was 15.2% for white subjects, 18.4% for Hispanics, 18.4% for Asians, and 20.2% for black subjects [41]. Clinical outcomes showed that patients with subclinical hypoxemia had higher serum creatinine and lactate levels than those without subclinical hypoxemia and higher sequential organ failure assessment (SOFA) scores and hospital mortality [41].

These studies consistently reported that skin color differences cause pulse oximeter measurement errors, and the errors are particularly pronounced in people with darker skin, increasing the risk of potential hypoxia, organ damage, and worse clinical outcomes. Such disparities occur since most of the data that manufacturers obtain to develop pulse oximeters were derived from white subjects [42].

Following these reports, the Food and Drug Administration (FDA) has announced that to test the minimum mean accuracy of pulse oximeters, the SpO_2_ readings must be compared with directly measured SaO_2_ levels between 70 and 100% [43]. The FDA approves the pulse oximeter’s accuracy if 66% of SpO_2_ values are within 2–3% of SaO_2_ values and about 95% of SpO_2_ values are within 4–6% of SaO_2_ values, emphasizing that SpO_2_ values do not always agree with SaO_2_ [43].

The pulse oximeter has become an essential monitoring device in clinical practice because it is a non-invasive and simple tool to measure oxygen saturation. However, it is fraught with potential health hazards due to misinterpretation of the numbers. Therefore, clinicians need to be aware of the limitations of pulse oximeters and make careful clinical decisions.

## Conclusion

NPPV has a well-established beneficial effect in acute exacerbations of COPD and cardiogenic pulmonary edema and probably reduces mortality in patients with acute hypoxemic respiratory failure. In COVID-19 respiratory failure, NPPV is favored over HFNC. HFNC may also reduce mortality in patients with acute hypoxemic respiratory failure and is recommended in recent guidelines. Questions remain regarding the best NPPV interface, the clinical impact of p-SILI, and the timing of and indications for intubation when NIV is started. All clinical practitioners use pulse oximetry despite its potential inaccuracies, particularly in patients with darker skin colors. Clinicians need to be aware of the unreliability of pulse oximeters to avoid exposing those patients to harmful clinical decision making.

## Data Availability

Not applicable.
